# Above- and below-ground functional trait coordination in the Neotropical understory genus *Costus*

**DOI:** 10.1093/aobpla/plab073

**Published:** 2021-12-02

**Authors:** Eleinis Ávila-Lovera, Gregory R Goldsmith, Kathleen M Kay, Jennifer L Funk

**Affiliations:** 1 Schmid College of Science and Technology, Chapman University, Orange, CA 92866, USA; 2 Department of Ecology and Evolutionary Biology, University of California, Santa Cruz, CA 95060, USA

**Keywords:** Ecophysiology, functional strategies, rhizome traits, specific root length, stem specific density, tropics, variance component analysis

## Abstract

The study of plant functional traits and variation among and within species can help illuminate functional coordination and trade-offs in key processes that allow plants to grow, reproduce and survive. We studied 20 leaf, above-ground stem, below-ground stem and fine-root traits of 17 *Costus* species from forests in Costa Rica and Panama to answer the following questions: (i) Do congeneric species show above-ground and below-ground trait coordination and trade-offs consistent with theory of resource acquisition and conservation? (ii) Is there correlated evolution among traits? (iii) Given the diversity of habitats over which *Costus* occurs, what is the relative contribution of site and species to trait variation? We performed a principal components analysis (PCA) to assess for the existence of a spectrum of trait variation and found that the first two PCs accounted for 21.4 % and 17.8 % of the total trait variation, respectively, with the first axis of variation being consistent with a continuum of resource-acquisitive and resource-conservative traits in water acquisition and use, and the second axis of variation being related to the leaf economics spectrum. Stomatal conductance was negatively related to both above-ground stem and rhizome specific density, and these relationships became stronger after accounting for evolutionary relatedness, indicating correlated evolution. Despite elevation and climatic differences among sites, high trait variation was ascribed to individuals rather than to sites. We conclude that *Costus* species present trait coordination and trade-offs that allow species to be categorized as having a resource-acquisitive or resource-conservative functional strategy, consistent with a whole-plant functional strategy with evident coordination and trade-offs between above-ground and below-ground function. Our results also show that herbaceous species and species with rhizomes tend to agree with trade-offs found in more species-rich comparisons.

## Teaser

The genus *Costus* comprises herbaceous species that inhabit sites that range from lowland to montane forests, from deep shade in the understory to high light gaps, and from dry forest edges to wet ravines and swamps. How do these varied environments shape plant functional traits? Despite this habitat diversity, we found that species and individuals, rather than site, explained the most trait variation. Species of *Costus* show coordination and trade-offs among traits consistent with resource-acquisitive and resource-conservative strategies consistent with other genera, and these strategies can co-occur within sites.

## Introduction

Functional traits are defined as any morphological, physiological or phenological characteristic that indirectly influences fitness through their effects on growth, reproduction and survival (Violle *et al.* 2007). However, traits do not usually work in isolation. Indeed, ecologists often use suites of correlated functional traits, i.e. plant strategies, to provide insights into the way that plants acquire, use and conserve resources (Reich *et al.* 2003; Wright *et al.* 2004; Zanne *et al.* 2010; Reich 2014). Resource-acquisitive strategies involve traits that allow for fast acquisition and use of resources (high turnover), which in turn results in fast growth rates and low capacity to tolerate stress. On the other hand, resource-conservative strategies involve traits that allow for slow use of resources and slow growth, but with increased capacity to tolerate stress. For instance, leaf traits related to carbon and nutrient economy have been found to covary among plant species worldwide resulting in a ‘leaf economics spectrum’ (LES) (Reich *et al.* 1997; Westoby *et al.* 2002; Wright *et al.* 2004). In this spectrum, leaves with high specific leaf area (SLA) and high nutrient concentration also have high photosynthetic and respiration rates, but a short lifespan (resource-acquisitive strategy), whereas leaves with low SLA and nutrient concentration also have low photosynthetic and respiration rates, but a long lifespan (resource-conservative strategy) (Reich *et al.* 1997; Wright *et al.* 2004). This economic spectrum has been extended to stems (Chave *et al.* 2009) and, with mixed support, to roots (Kong *et al.* 2015, 2019; Roumet *et al.* 2016). The existence of spectra of variation within organs suggests that coordination (positive covariance) and trade-offs (negative covariance) among traits limit organ function, and likely whole-plant function.

Despite the importance of below-ground processes on plant physiology and performance (Laliberté 2017), below-ground traits have only recently been incorporated into plant functional strategy frameworks (McCormack *et al.* 2012; Mommer and Weemstra 2012; Weemstra *et al.* 2016). Root traits that allow plants to acquire large amounts of water and nutrients, such as high root length density (cm root cm^−3^ soil) or specific root length (SRL; m g^−1^), should be beneficial in resource-rich environments, whereas traits that allow plants to avoid water stress by accessing more stable sources of water and restricting resource loss (or encouraging conservation), such as high root diameter or root tissue density (RTD; g cm^−3^), should be beneficial in resource-poor environments (Bowsher *et al.* 2016). Specific root length is suggested to be the below-ground analogue to SLA, but SRL is often orthogonal to the main axis of root variation usually formed by root diameter and RTD (Freschet *et al.* 2010; Liu *et al.* 2010; Bowsher *et al.* 2016; Kramer-Walter *et al.* 2016; Weemstra *et al.* 2016), complicating efforts to identify a single axis characterizing below-ground function that aligns with above-ground function (Reich 2014). More recently, the inclusion of mycorrhiza (e.g. mycorrhizal colonization) in root functional trait frameworks has shown the existence of a fungal collaboration gradient that dominates the root economic spectrum in a large data set of species (McCormack and Iversen 2019; Bergmann *et al.* 2020; Weigelt *et al.* 2021). However, the question remains as to what extent plants align their above-ground and below-ground traits, i.e. are traits coordinated across organs reflecting a single unified whole-plant functional strategy?

Other below-ground traits have been completely left out from recent plant functional strategy frameworks. For example, some plant species use below-ground stems (i.e. rhizomes) as important anchoring structures, for carbohydrate and water storage, and for vegetative reproduction. Traits such as rhizome water content (RhWC; %) and rhizome specific density (RhSD; g cm^−3^) provide information on water storage capacity or investment in structural carbon in rhizomes. Evaluating rhizome traits of perennial herbs can give us insights into the growth strategy of these plants. We expect that species with below-ground resource-conservative traits would also have low RhWC and high RhSD, indicating a greater investment in structural carbon rather than in water storage, with opposite values of traits corresponding to resource-acquisitive. These rhizome traits would also align with fine-root traits, and above-ground traits, if a whole-plant economic spectrum does exist (Reich 2014).

We studied closely related species from the genus *Costus* because (i) species co-occur at multiple sites, (ii) a well-resolved phylogeny exists (Vargas *et al.* 2021) and (iii) they have speciated rapidly in recent history, giving opportunities to study traits that have recently evolved. By using congeneric species, we reduced the effect of large divergence patterns (e.g. across families or life forms) on observed trait values.

Given the potential importance for above-ground and below-ground relationships in determining whole-plant functional strategies, we studied a group of closely related tropical species in the genus *Costus* living in contrasting habitats to answer the following questions: (i) Do congeneric species show above-ground and below-ground trait coordination and trade-offs consistent with theory of resource acquisition and conservation? (ii) Is there correlated evolution among traits? (iii) Given the diversity of habitats over which *Costus* occurs, what is the relative contribution of site and species to trait variation? Given the habitat variability across sites, and the morphological differences among species living at the same site, we hypothesized that *Costus* will show a diversity of strategies (i.e. combination of traits), ranging from resource-acquisitive to resource-conservative strategies that matches their habitats. For example, species in wet habitats are expected to have traits that allow for greater water use, such as low rhizome and stem specific density and high stomatal conductance. We also hypothesized that species identity plays a significant role in explaining trait variation. Research to date on tropical plant functional traits has largely been confined to woody species and to the context of community assembly (Kraft *et al.* 2008), with little work on how functional traits and trade-offs can help understand the physiological mechanisms by which herbaceous species respond to environmental variation.

## Methods

### Study sites

**Figure 1.**
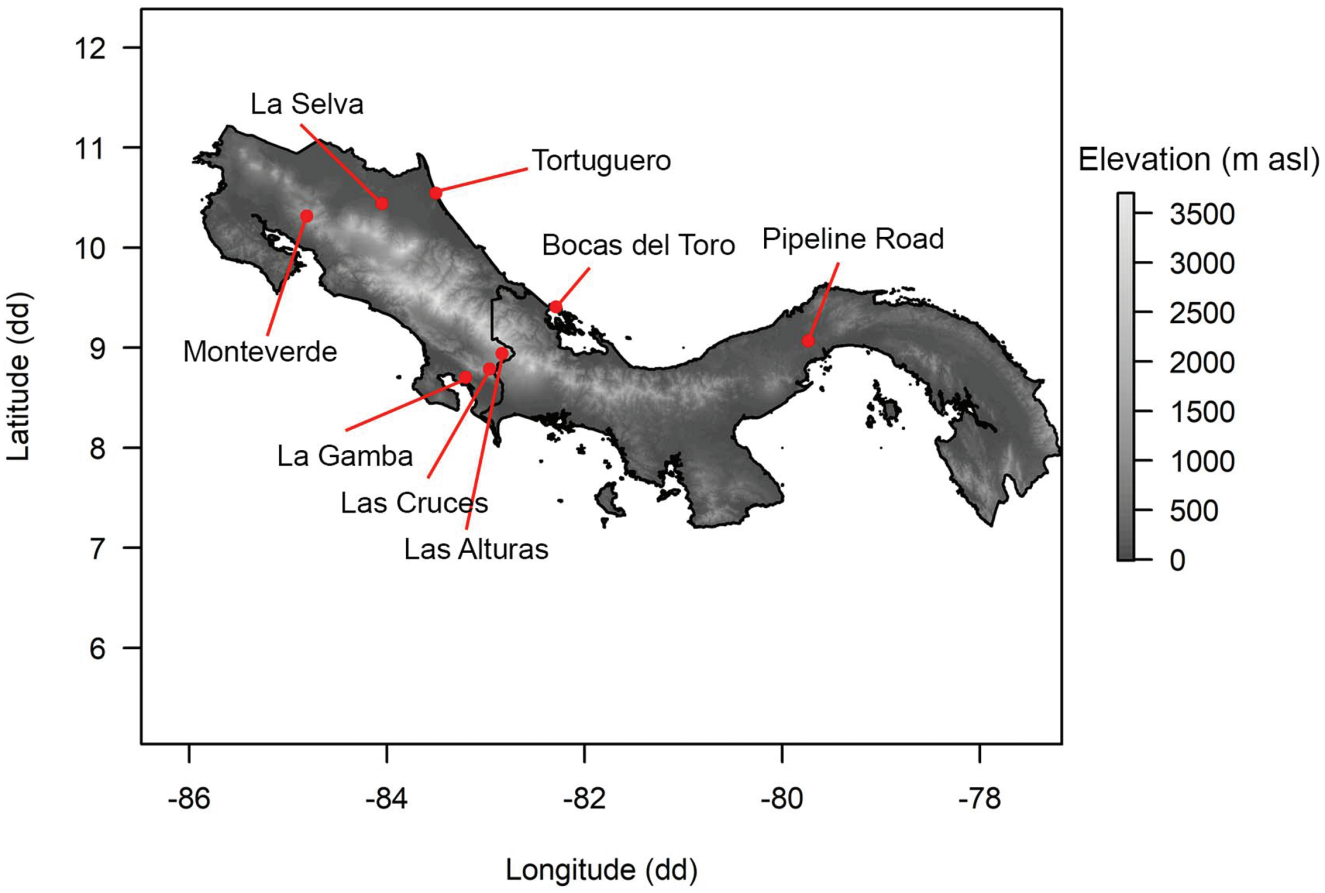
Map of Costa Rica and Panama showing the geographic location of the eight field sites. Shading corresponds to elevation (m asl).

We measured leaf, above-ground stem, below-ground stem (rhizome from now on) and fine-root traits on individual plants of 17 species of *Costus* in six sites in Costa Rica and two sites in Panama (6 of these 17 species were present at more than one site; Table 1) during the rainy season. Field sites varied in elevation (Fig. 1), which affects mean annual temperature (MAT) and precipitation (MAP) as well as precipitation seasonality. We used the latitude and longitude of the sampled individuals to download bioclimatic variables from WorldClim 2.0 (Fick and Hijmans 2017) and then averaged by species and sites. Lowland wet forests have high MAT and MAP and low precipitation seasonality, whereas highland wet montane and pre-montane forests have relatively lower MAT and MAP and high precipitation seasonality (E. Ávila-Lovera *et al*., submitted for publication). Lowland seasonal forests have a more pronounced dry season than lowland wet forests (**see****Supporting Information—Table S1** for more bioclimatic data of field sites). Permit information can be found in Supporting Information—Notes S1.

**Table 1.** List of species studied, abbreviation use in figures, sites where they are present, elevation and habitat type. BDT: Bocas del Toro (Panama), LA: Las Alturas (Costa Rica), LC: Las Cruces (Costa Rica), LG: La Gamba (Costa Rica), LS: La Selva (Costa Rica), MV: Monteverde (Costa Rica), PLR: Pipeline Road (Panama), TG: Tortuguero (Costa Rica).

Species	Abbreviation	Site	Elevation (m asl)	Habitat type
*C.* aff*. wilsonii*	aff.wils	MV	1519.3	Montane forest, streams
*C. alleni*	alle	PLR	113.5	Wet forest, deep shade
*C. bracteatus*	brac	LS	77.5	Wet forest
		TG	12.3	Wet forest
*C. guanaiensis* var. *macrostobilus*	guan	PLR	69.2	Seasonal forest
*C. laevis*	laev	LC	1216.8	Pre-montane forest, streams
		LG	113.2	Wet forest
		LS	61.5	Wet forest
		PLR	92.0	Seasonal forest
		TG	20.0	Wet forest
*C. lima*	lima	LG	82.0	Wet forest, riverine
*C. malortieanus*	malo	LS	56.0	Wet forest
*C. montanus*	mont	MV	1569.3	Montane forest
*C. osae*	osae	LG	122.2	Wet forest, streams
*C. plicatus*	plic	LG	112.2	Wet forest, riverine
*C. pulverulentus*	pulv	LG	130.5	Wet forest
		LS	68.8	Wet forest, treefall gaps
		PLR	73.0	Seasonal forest
		TG	16.4	Wet forest
*C. ricus*	ricu	LG	211.25	Wet forest
*C. scaber*	scab	LG	227.7	Wet forest
		LS	78.4	Wet forest, streams
		PLR	74.8	Seasonal forest
		TG	26.3	Wet forest
*C. stenophyllus*	sten	LG	180.2	Wet forest
*C. villosissimus*	vill	PLR	70.0	Seasonal forest, forest edges
*C. wilsonii*	wils	LA	1559.0	Montane forest
		LC	1216.8	Pre-montane forest
*C. woodsonii*	wood	BDT	0	Beach
		TG	3.8	Beach

### Study species

The genus *Costus* (Costaceae) comprises approximately 60 species in the Neotropics, and it occupies habitats that range from lowland to montane forests, from deep shade understory to high light gaps and from dry forest edges to ravines and swamps (Kay *et al.* 2005). Therefore, it is an interesting group to study plant trait coordination and trade-offs. Furthermore, species in the genus have the ability to reproduce vegetatively via rhizomes, which also store water, carbohydrates and nutrients (Klimešová *et al.* 2018).

We sampled adult individuals of species of the genus *Costus* during the wet seasons of 2018 (July, Costa Rica) and 2019 (June, Panama). For all traits measured, we aimed to sample six individuals per species per site (Table 1; **see****Supporting Information—Table S1**), but could not do it for the following species: *C. bracteatus* and *C. laevis* in Tortuguero (*n* = 3 each), and *C. lima* (*n* = 2) and *C. ricus* (*n* = 4) in La Gamba.

### Plant functional traits

We chose plant functional traits related to the carbon and water economy of plants, that vary in response to environmental conditions, including precipitation and temperature regime (E. Ávila-Lovera *et al*., submitted for publication), and that are commonly measured to allow for comparison with other studies. We also included less common traits, such as RhWC and RhSD, as species in the *Costus* genus have perennating rhizomes that are important for the life of the plants. We studied a total of 20 traits among leaves, above-ground stems, rhizomes and fine roots **[see****Supporting Information—Table S1**; **Fig. S3–S22****]**.

#### Above-ground traits.

All leaf traits were measured in one fully expanded leaf per individual, usually from the fourth–sixth node of the plant to standardize leaf age across individuals. We chose to include traits that could vary with light environment and water and nutrient availability, the so-called economic traits, and followed standard protocols (Cornelissen *et al.* 2003; Pérez-Harguindeguy *et al.* 2013).

Leaf relative chlorophyll content (Chl; SPAD units) and maximum stomatal conductance (*g*_s_; mmol m^−2^ s^−1^) were measured *in situ* between 0730 and 1200 h preferably on rainless days. Chlorophyll content was measured using a digital chlorophyll meter (SPAD 502, Konica Minolta Sensing Inc., Japan), whereas *g*_s_ was measured with a steady-state leaf porometer (SC-1, Meter Environment, USA). After these measurements were taken, the leaf, including the petiole, was collected, placed in a zip lock bag and transported to the lab for further processing.

In the lab, we measured leaf thickness (LT; mm) in the middle portion of the leaf (avoiding major veins) using a digital micrometer (Mitutoyo IP65, Global Industrial, Port Washington, NY, USA); then, the whole leaf (lamina + petiole) was photographed against a white background including a ruler. We determined leaf size as leaf area (LA; cm^2^) using ImageJ software (Rasband 1997). The leaf was then weighed whole to obtain leaf fresh mass and dried at 60 °C for 72 h to obtain leaf dry mass. From these variables we calculated multiple leaf traits: leaf dry matter content (LDMC; mg g^−1^), calculated as leaf dry mass divided by leaf fresh mass, i.e. what proportion of the whole leaf is not water; SLA (cm^2^ g^−1^), calculated as LA divided by leaf dry mass; and finally, we calculated two traits, lamina dry mass to petiole dry mass ratio (LM:PM ratio; g g^−1^) and leaf area to petiole dry mass ratio (LA:PM ratio; cm^−2^ g^−1^), that have been previously studied in palms and heliconias as a measure of the costs of leaf mass support (Chazdon 1986; Rundel *et al.* 1998).

Dry leaf samples were ground to a fine powder using a mill (Wiley mini-mill, Thomas Scientific, Swedesboro, NJ, USA) and analysed for phosphorus (P; %) and potassium (K; %) concentrations at the Analytical Laboratory of the University of California, Davis (UC Davis). Ground leaf samples were also sent to the UC Davis Stable Isotope Facility for determination of carbon isotopic composition (δ ^13^C; ‰), carbon concentration (C; %), nitrogen isotopic composition (δ ^15^N; ‰) and nitrogen concentration (N; %). Values of δ ^13^C were standardized against Vienna Pee Dee Belemnite.

From the same plant sampled for leaf traits, we collected a *c.* 10-cm-long stem sample subtending the leaf previously sampled, which was placed in a zip lock bag and transported to the lab for further processing. From the collected stem, we sectioned a 2-cm-long piece and measured its fresh volume using the water mass displacement method (De Guzman *et al.* 2017). Then, the stem sample was dried at 60 °C for 72 h to obtain stem dry mass. We calculated stem specific density (SSD; g cm^−3^) as stem dry mass divided by stem fresh volume.

#### Below-ground traits.

Rhizomes with attached roots were dug up, placed in a zip lock bag and transported to the lab for further processing. Rhizomes were washed, and a portion was sectioned, blotted dry and its fresh mass measured. Fresh volume was determined as in stems, and the rhizome portion was dried at 60 °C for 72 h to obtain rhizome dry mass. We calculated RhWC (%) as rhizome water mass divided by rhizome fresh mass and multiplied by 100, and RhSD (g cm^−3^) was calculated as rhizome dry mass divided by rhizome fresh volume.

Fine roots (<2 mm thick) that were attached to the rhizomes via coarse roots were collected from 0 to 10 cm soil depth. These fine roots were measured for length and dried at 60 °C for 72 h to obtain fine-root dry mass. Specific root length (m g^−1^) was calculated as fine-root length divided by fine-root dry mass. Photos of the fresh roots were taken and fine-root diameter (FRD; mm) was measured using ImageJ. Length and diameter of fine roots were then used to calculate fine-root volume, and dry mass and volume were used to calculate RTD (g cm^−3^). We could not obtain fine-root traits for samples in Panama for logistic reasons.

### Statistical analyses

We performed one principal components analysis (PCA) for above-ground and below-ground traits to extract the main axes of variation using both individual data points and species means across sites and the ‘prcomp’ function in R v.3.6.6 (R Core Team 2020). Traits were standardized to mean 0 and standard deviation 1 before running the analysis. We tested for phylogenetic signal in traits using species means across sites (H_0_ of Blomberg’s *K* = 0, significance at *P* < 0.05) and using the ‘phylosig’ function of the ‘phytools’ package in R (Revell 2012, 2019), and found no phylogenetic signal for any trait **[see****Supporting Information—Table S2****]**; thus, we did not include phylogenetic information in the PCAs. These results contrast with what has been found for some root traits in a large data set (Valverde-Barrantes *et al.* 2017).

To test for pairwise correlations among traits, we performed an analysis on the species means across sites using the ‘rcorr’ function of the ‘Hmisc’ package in R, and the plots were performed with the function ‘corrplot’ from the ‘corrplot’ package in R (Wei *et al.* 2021). To infer correlated evolution of traits, we estimated phylogenetic independent contrasts (PICs) of species means across sites using the function ‘pic’ of the ‘ape’ package in R (Paradis *et al.* 2004, 2018) and a phylogenetic tree **[see****Supporting Information—Fig. S1****]** constructed from a larger *Costus* phylogeny publicly available (Vargas *et al.* 2021). Finally, we ran a correlation analysis using the PICs. We decided not to correct for multiple testing, as recommended by Moran (2003).

To determine the contribution of site, species and individuals to the observed trait variation, we performed a variance component analysis. We ran a general linear mixed-effects model using the ‘lmer’ function from the ‘lme4’ package (Bates *et al.* 2015, 2020), to determine the proportion of variance explained by the three factors: site, species and individuals. The model used the raw trait data, not mean values.

## Results

**Figure 2.**
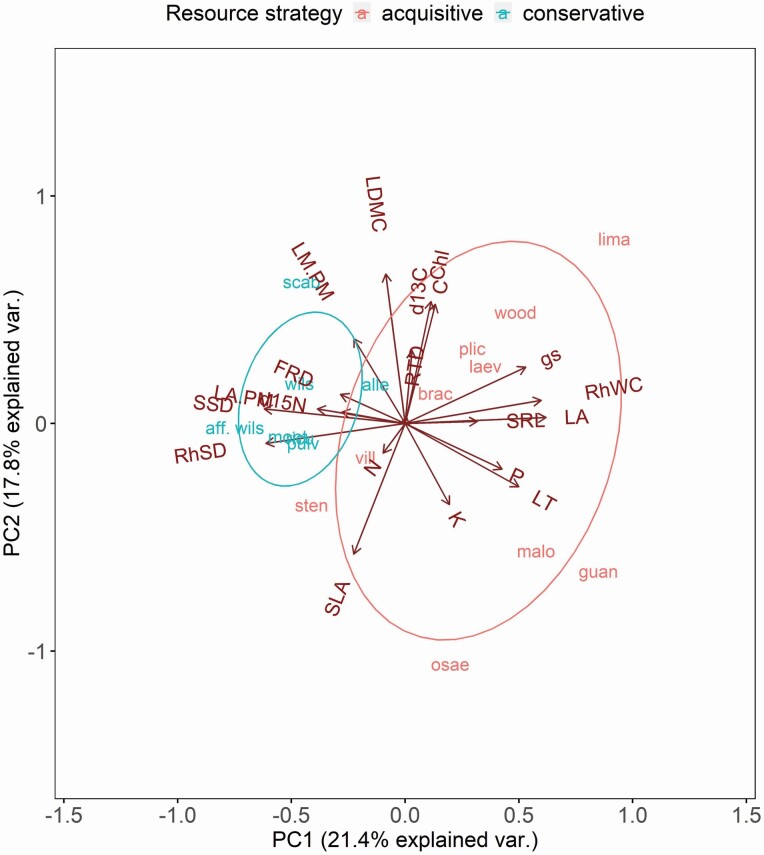
Principal components analysis (PCA) biplot of the studied functional traits. Groupings denote species with resource-acquisition or resource-conservation strategies. Species are abbreviated as shown in Table 1. Chl: chlorophyll concentration; *g*_s_: stomatal conductance; LT: leaf thickness; LA: leaf area; LDMC: leaf dry matter content; SLA: specific leaf area; LM:PM: lamina dry mass to petiole dry mass ratio; LA:PM: leaf area to petiole dry mass ratio; P: leaf phosphorus concentration; K: leaf potassium concentration; δ ^13^C: leaf carbon isotopic composition; C: leaf carbon concentration; δ ^15^N: leaf nitrogen isotopic composition; N: leaf nitrogen concentration; SSD: stem specific density; RhWC: rhizome water content; RhSD: rhizome specific density; SRL: specific root length; FRD: fine-root diameter; RTD: root tissue density.

PCAs performed on species means across sites and on individual plant values were qualitatively similar; hence, we only present and discuss the former here (the latter can be found in **Supporting Information—Fig. S2**). The first two PC axes together explained 39.2 % of the total variation, with PC1 explaining 21.4 % and PC2 explaining 17.8 % (Fig. 2; Table 2). PC1 captured a trade-off between resource acquisition and conservation of water: species with a resource-acquisitive strategy had high LA, *g*_s_, LT, P and RhWC mostly indicating high water use; whereas species with a resource-conservative strategy had high LA:PM, SSD and RhSD mostly indicating low water use and transport (Fig. 2). PC2 explained a similar amount of variation as PC1, with a resource-conservation versus resource-acquisition trade-off that was in accordance with the leaf economic spectrum: Chl, LDMC, δ ^13^C and C loaded positively with PC2 indicating high investment in structure, and SLA and K loaded negatively with PC2 indicating high capacity for leaf photosynthesis (Fig. 2). Interestingly, most below-ground traits, especially fine-root traits, strongly loaded with PC3, being orthogonal to the rest of the traits (Table 2).

**Table 2.** Results from the PCA analysis on species means, including the eigenvalue of the first three PCs, the percent of total variance explained by the first three PCs and the cumulative variance explained. Trait loadings are also included, where bolded values indicate the highest loading of the trait among the three first PC axes.

	PC1	PC2	PC3
Eigenvalue	4.28	3.56	3.14
Percent of total variance explained (%)	21.4	17.8	15.7
Cumulative variance explained (%)	21.4	39.2	54.9
Trait loadings			
Above-ground traits			
Chlorophyll concentration	0.14	**0.73**	0.32
Stomatal conductance	**0.66**	0.34	0.14
Leaf thickness	**0.62**	−0.38	−0.25
Leaf area	**0.77**	0.04	−0.24
Leaf dry matter content	−0.11	**0.89**	−0.13
Specific leaf area	−0.28	**−0.78**	0.31
Leaf mass to petiole mass ratio	−0.28	**0.51**	−0.12
Leaf area to petiole mass ratio	**−0.48**	0.09	0.13
Phosphorus concentration	**0.53**	−0.27	0.43
Potassium concentration	0.24	**−0.48**	−0.19
Carbon isotopic composition	0.04	**0.44**	−0.11
Carbon concentration	0.16	**0.71**	−0.03
Nitrogen isotopic composition	**−0.34**	0.07	0.32
Nitrogen concentration	−0.12	−0.18	**0.74**
Stem specific density	**−0.77**	0.09	−0.11
Below-ground traits			
Rhizome water content	**0.74**	0.14	0.53
Rhizome specific density	**−0.76**	−0.12	−0.53
Specific root length	0.39	0.01	**−0.78**
Fine-root diameter	−0.35	0.17	**0.69**
Root tissue density	0.01	0.04	**0.45**

**Figure 3.**
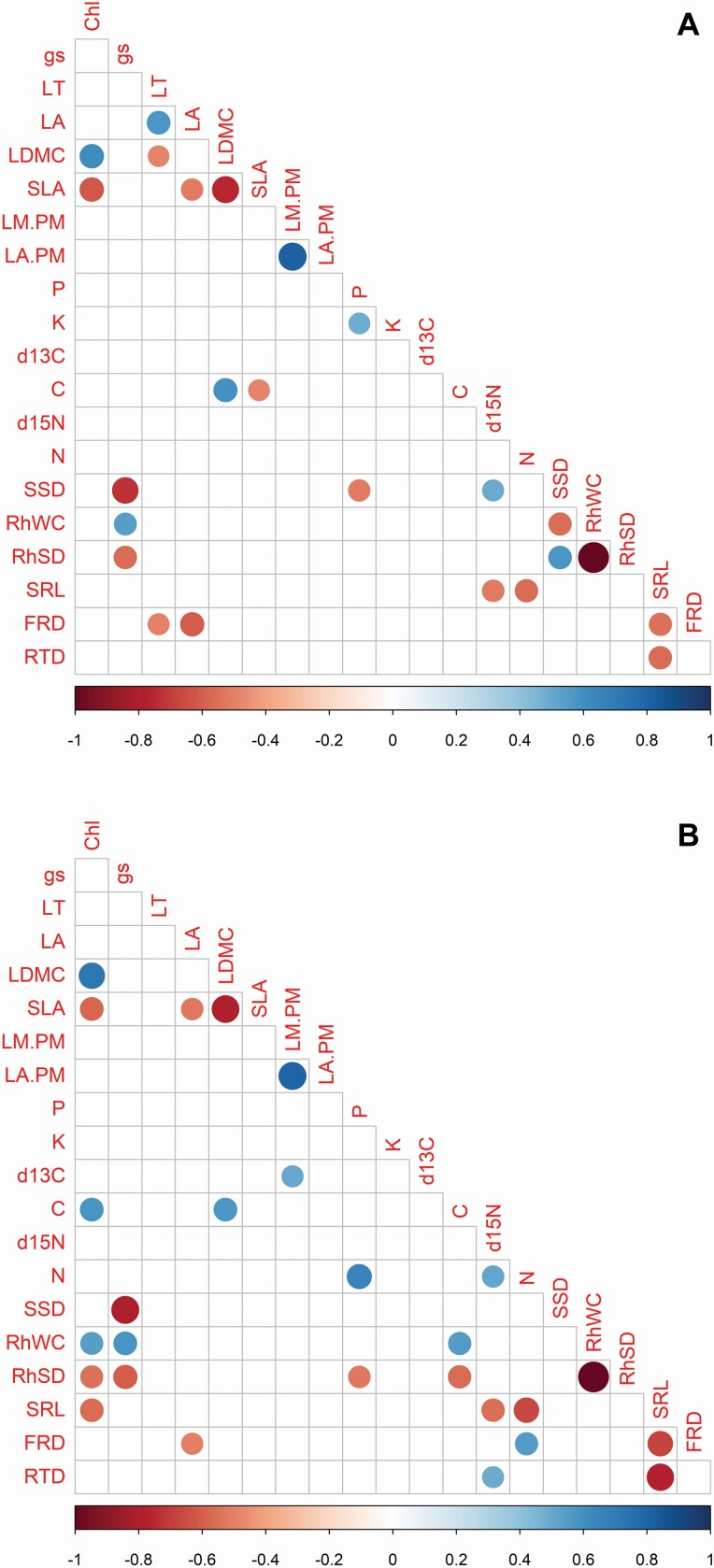
Correlation plots showing significant correlations only (*P* < 0.05). (A) Cross-species correlations. (B) Correlations using phylogenetic contrasts. Chl: chlorophyll concentration; *g*_s_: stomatal conductance; LT: leaf thickness; LA: leaf area; LDMC: leaf dry matter content; SLA: specific leaf area; LM:PM: lamina dry mass to petiole dry mass ratio; LA:PM: leaf area to petiole dry mass ratio; P: leaf phosphorus concentration; K: leaf potassium concentration; δ ^13^C: leaf carbon isotopic composition; C: leaf carbon concentration; δ ^15^N: leaf nitrogen isotopic composition; N: leaf nitrogen concentration; SSD: stem specific density; RhWC: rhizome water content; RhSD: rhizome specific density; SRL: specific root length; FRD: fine-root diameter; RTD: root tissue density.

**Figure 4.**
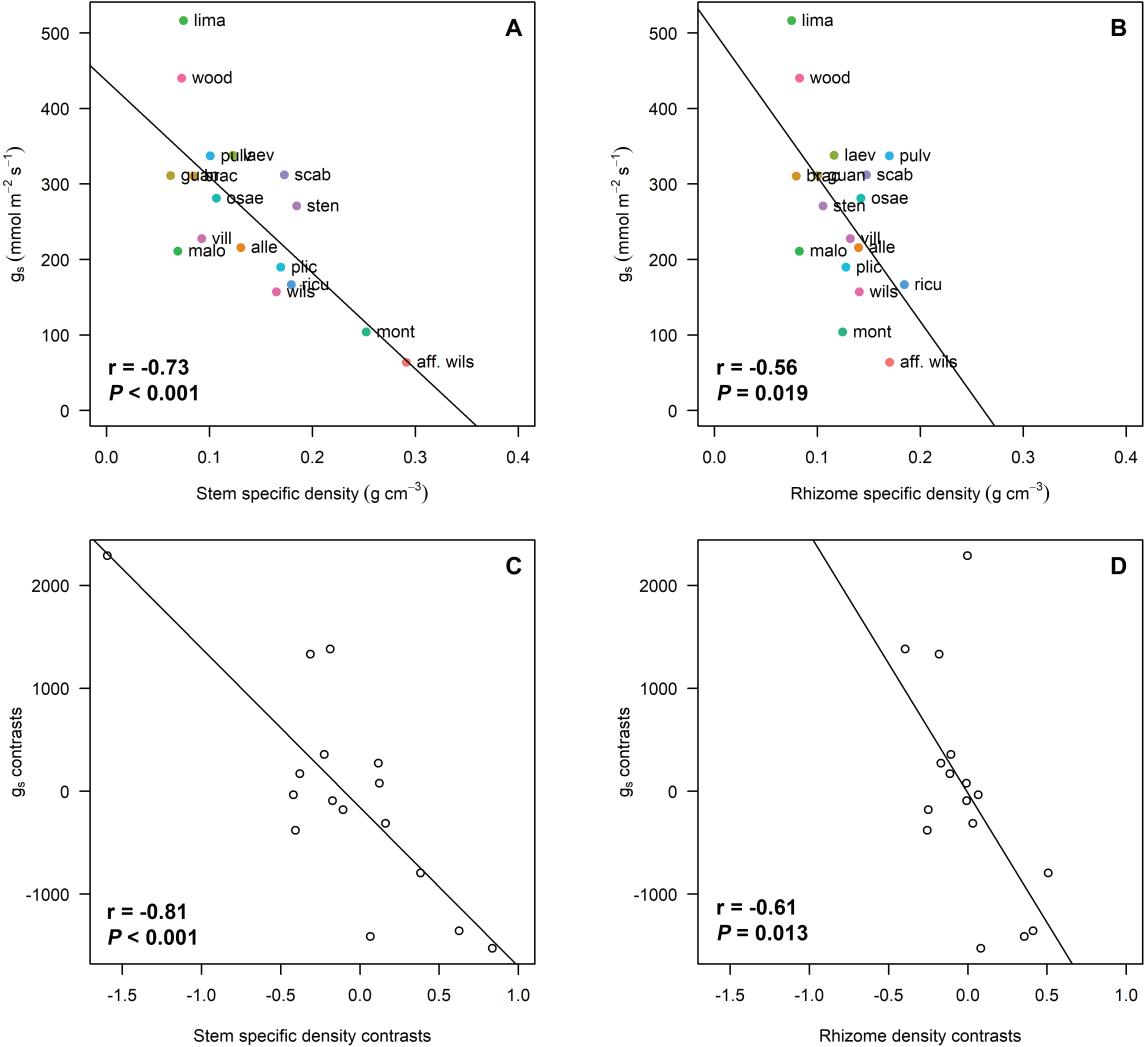
(A) Cross-species correlation between stomatal conductance and stem specific density and (B) between stomatal conductance and rhizome specific density. (C) Correlation between stomatal conductance contrasts and stem specific density contrasts, and (D) stomatal conductance contrasts and rhizome specific density contrasts. Contrasts were calculated as the difference between trait values of sister species divided by branch length. Trend line is included when correlations were significant. Species are abbreviated as shown in Table 1.

The pairwise correlations supported the relationships found among traits in the PCA (see all results in **Supporting Information—Table S3**), for example, LA and LT were positively related with each other (Fig. 3A), whereas *g*_s_ was negatively related to SSD (Figs 3A and 4A). Similarly, *g*_s_ was negatively related to RhSD (Figs 3A and 4B) and positively related to RhWC (Fig. 3A). Furthermore, when correlations were performed on the PICs, the number of significant correlations increased from 24 to 27 and the strength of most correlations (*r-*value) increased as well (Fig. 3). Some interesting correlations using both species means and PICs were between: (i) leaf size and thickness (Fig. 3); (ii) *g*_s_ and structural above-ground stem and rhizome traits: SSD, RhWC and RhSD (Figs 3 and 4); (iii) SRL and leaf N and δ ^15^N (Fig. 3); and (iv) SRL and RTD (Fig. 3).

**Figure 5.**
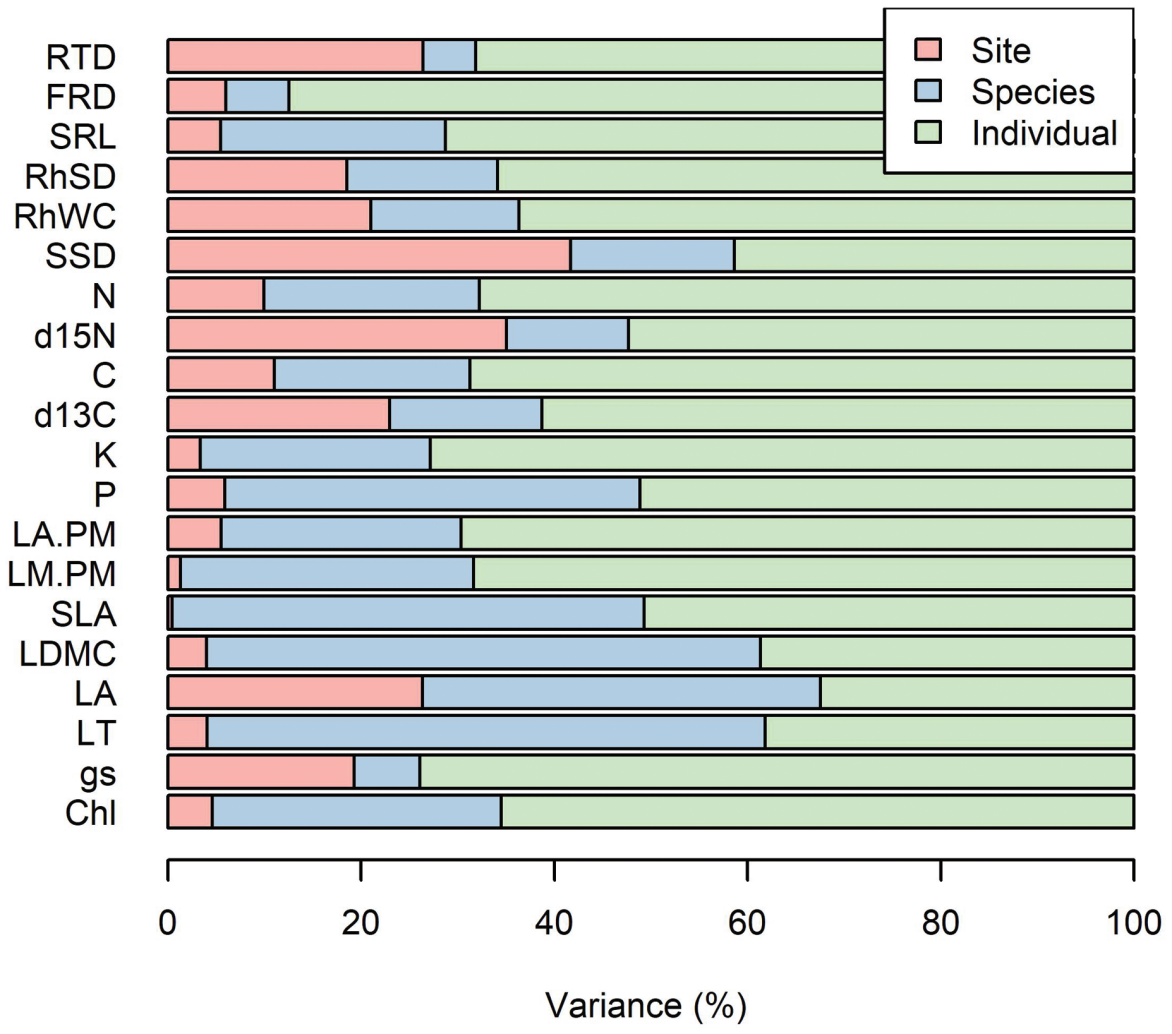
Variance component analysis of the traits studied. We partitioned the total trait variation into three levels: sites, species and individuals. Chl: chlorophyll concentration; *g*_s_: stomatal conductance; LT: leaf thickness; LA: leaf area; LDMC: leaf dry matter content; SLA: specific leaf area; LM:PM: lamina dry mass to petiole dry mass ratio; LA:PM: leaf area to petiole dry mass ratio; P: leaf phosphorus concentration; K: leaf potassium concentration; δ ^13^C: leaf carbon isotopic composition; C: leaf carbon concentration; δ ^15^N: leaf nitrogen isotopic composition; N: leaf nitrogen concentration; SSD: stem specific density; RhWC: rhizome water content; RhSD: rhizome specific density; SRL: specific root length; FRD: fine-root diameter;, RTD: root tissue density.

The variance component analysis showed that there was substantial trait variation explained by individuals: 32–87 % of total trait variation compared to 5–58 % explained by species, and 0.4–42 % explained by sites (Fig. 5). Individual variation was particularly high in FRD and *g*_s_ (Fig. 5). Remarkably, site only explained a relatively high proportion of variance in RTD (26 %), leaf δ ^15^N (35 %) and SSD (42 %) (Fig. 5). Specific leaf area was the trait with the lowest variation ascribed to site (Fig. 5), despite its important role describing the leaf economic spectrum.

## Discussion

We studied 20 leaf, above-ground stem, rhizome and fine-root traits of 17 *Costus* species in eight sites that span lowland seasonal and wet forests to pre-montane and montane wet forests. We found evidence for trait coordination and trade-offs among functional traits, as well as correlated evolution. Furthermore, trait variation ascribed to individuals was high across all traits measured, indicating a high contribution of individual variation to total within-genus variation for *Costus*.

We found coordination and trade-offs among traits that are consistent with two distinct axes of resource acquisition and conservation related to different functions. The first two axes of the PCA roughly explained a similar amount of the total trait variation, precluding the existence of a single major axis of variation as observed in some studies (Freschet *et al.* 2010; Liu *et al.* 2010). For example, PC1 showed coordination and trade-offs among traits, both above-ground and below-ground, related to water acquisition, use and movement. On the other hand, traits loading on PC2 were consistent with the leaf economic spectrum, with some species having high leaf C and LDMC, aligning with a resource-conservation strategy, whereas others had low LDMC and high nutrient concentrations, corresponding with a resource-acquisition strategy. Taken together, these results are consistent with the global plant economic spectrum (Reich 2014; Díaz *et al.* 2016) and support the idea of a unified whole-plant functional strategy. Interestingly, fine-root traits loaded strongly with the third PC, not being related to the first two axes of variation.

Few studies have examined below-ground traits and how they relate to above-ground traits and results from these studies are often contradictory. For example, some studies found no coordination between leaf, stem and root traits (Fortunel *et al.* 2012; Bowsher *et al.* 2016; Silva *et al.* 2017), whereas others found leaf-root functional coordination (Freschet *et al.* 2010; Liu *et al.* 2010). In our system, rhizomes provide structural support and water transport to above-ground organs (Maas 1972) and thus may be critical for herbaceous plants to achieve tall heights (e.g. 2 m in *C. montanus*) given their lack of woody tissue; hence, they play an important role in plant functioning. We found that rhizome traits related to above-ground traits loading on PC1, but were orthogonal to SRL, indicating that below-ground function in these species may be multidimensional, as has been previously found in a review of tree species (Weemstra *et al.* 2016), seedlings of temperate tree species (Kramer-Walter *et al.* 2016) and temperate herbaceous plants (Zhou *et al.* 2018). Being perennial organs, rhizomes perform multiple functions: they provide support for aerial shoots, serve as carbohydrate storage organs and are a mean for vegetative reproduction (clonality); as anatomical stems, they hydraulically connect roots and aerial shoots. The importance of rhizomes for the life of *Costus* plants is evidence in their coordination with above-ground function.

One reason for the no coordination between above-ground and fine-root traits is that multiple combinations of these traits can be favoured in the Neotropical forests sampled. A previous study on trait–environment relationships using the same species studied here suggested that few functional traits respond to environmental variation (E. Ávila-Lovera *et al*., submitted for publication). In the current study, for example, closely related *C. osae* and *C. lima* are found in La Gamba, Costa Rica, and experience similar macroclimate conditions (MAT and MAP). However, these species have different suites of above-ground traits: *C. osae* has low LDMC and high nutrients (K and P), whereas *C. lima* has high leaf C, low N and *g*_s_, albeit having similar values of RhWC, RhSD and SRL. These results may indicate adaptation to different light microhabitats: *C. osae* occurs in shady ravines, where there is high water availability (low LDMC is favoured) but low light availability (where leaf nutrients can enhance photosynthetic activity), and *C. lima* is found in sun-exposed habitats characterized by both high water and light availability (high *g*_s_ is favoured).

The relatively weak coordination between above-ground and below-ground traits in these herbaceous plants contrasts with those from woody congeneric species at higher latitudes, such as aspen (Hajek *et al.* 2013) and oak (Cavender-Bares *et al.* 2004, 2020), and community-level studies (Withington *et al.* 2006; Liu *et al.* 2010), where a clear coordination between below-ground and above-ground function exists. The only study performed in the tropics that we are aware of in which above-ground and below-ground function was studied, also found little coupling between above- and below-ground functional traits in dry forest seedlings (Arrieta-González *et al.* 2021), and the authors suggested the existence of multiple strategies to cope with water deficit. When comparing our results to those found at higher latitudes, seasonality experienced by the woody species rather than difference in growth form may explain such differences, such that only certain combinations of traits may be successful in highly seasonal environments at high latitudes (but see Pivovaroff *et al.* 2016). The limited coordination among below-ground traits, but also among below-ground and above-ground traits may be due to the multiple functions that below-ground organs perform, and hence a single main axis of trait variation may be precluded altogether. Variation in plant form and function within and among species creates the basis for species co-existence, plasticity and evolvability (Silva *et al.* 2017). This way, multiple combinations of traits that lead to different strategies among congeneric species may facilitate their co-existence within highly diverse plant communities (Bruelheide *et al.* 2018), especially in the Neotropics.

Correlations can sometimes better evidence the co-variation nature of traits among organs. Even though fine-root traits were strongly related to each other, they were rarely related to other traits, below- or above-ground. One of the few notable relationships was between SRL and both leaf δ ^15^N and N, where species with high SRL also had low leaf δ ^15^N and N. This is an interesting combination of traits given that high SRL indicates fast acquisition of water and nutrients, which can be beneficial if paired with high rates of carbon acquisition mediated by high N concentration. However, in our data set, species with low SRL had high leaf N and leaf δ ^15^N. Low SRL indicates low ability to explore soil for water and nutrient sources; however, these species have leaves with high N concentration, characteristics of the fast-return end of the LES. One aspect of below-ground function that we did not explore and could explain these trait–trait associations is the capacity of plants to form associations with mycorrhizal fungi. It has been recently reported that the fungal collaboration gradient dominates the root economic spectrum in a large data set of species (Bergmann *et al.* 2020). More work is needed to unravel this mystery; it is possible that additional traits not measured here (e.g. relative growth rate, whole-plant biomass allocation patterns, rates of nutrient uptake) may shed light on these seemingly contradictory relationships.

Correlated evolution among traits within groups of closely related species is common (Santiago and Kim 2009; Kembel and Cahill 2011; Liu *et al.* 2012; Savage and Cavender-Bares 2012; Sedio *et al.* 2012; Zhang *et al.* 2014; Bruy *et al.* 2018; Gallaher *et al.* 2019). We found correlated evolution among LA and LT. Leaf area determines the capacity to intercept light (Díaz *et al.* 2016) and has known impacts on leaf energy and water balance (Cornelissen *et al.* 2003). In *Costus*, species with large leaves, and likely high competitive ability, also have thick leaves of high succulence. High succulence allows for greater metabolite storage and has implications for structure and defence (Gutterman and Chauser-Volfson 2000; Mason *et al.* 2016). We also found correlated evolution among some above-ground and below-ground traits, which supports the hypothesis of the existence of the plant economic spectrum (Freschet *et al.* 2010; Reich 2014). For example, *g*_s_ was negatively correlated with SSD and RhSD, and positively related to RhWC, indicating that these traits evolved together and that species with high stomatal opening and profligate water use have low structural investment in above-ground stems and rhizomes. This low structural investment may indicate short lifespan but may promote high hydraulic efficiency (anatomical work to test this relationship is underway). Finally, SRL, our fine-root trait analogous to SLA of the LES, only evolved in a correlated fashion with chlorophyll concentration and leaf nutrients, but in the opposite direction to what it is expected: species with high SRL had low leaf chlorophyll concentration, leaf N and δ ^15^N. This unexpected relationship between SRL and leaf N requires further study in environments where water and nutrient availability can be controlled and independently modified.

Plant functional traits usually vary as a function of climate (Cavender-Bares *et al.* 2004; Wright *et al.* 2004; Violle *et al.* 2007; Moles *et al.* 2014; Mitchell *et al.* 2015; Blonder *et al.* 2017). In our study however, some traits, such as leaf (LT, LA, LDMC and SLA) and rhizome structural traits (RhSD), had low percent of variation ascribed to site. Even within a single genus, we expected trait variation ascribed to sites given the wide macroclimatic conditions experienced by species in sites that differ in elevation **[see****Supporting Information—Table S1****]**, and the fact that some traits do respond to climate variation in these *Costus* species (E. Ávila-Lovera *et al.* submitted for publication). However, our results align with other studies that have found low variation due to site in SLA, LA and LT in tropical riparian plant communities (Liu *et al.* 2018). That there was no variation due to site indicates that (i) those structural traits in the genus *Costus* are less labile than other traits (i.e. physiological and nutrient traits), indicating that they have lower capacity to be adjusted during the course of the plant lifetime (Scheiner 1993), or (ii) that there are phylogenetic constrains in those traits, which is further supported by slightly higher Blomberg’s *K* values for rhizome structural traits (RhWC and RhSD) than for other traits **[see****Supporting Information—Table S2****]**. Even though we found little trait variation that can be ascribed to site, it is important to note that there is habitat variation within sites, and species may be responding to this variation, rather than macroclimatic conditions. Future studies in which habitat variation can be better characterized will add to our understanding of trait variation in this genus.

Across all traits, a high proportion of total trait variation was ascribed to individual variation. This is consistent with results from a recent meta-analysis that showed a high contribution of intraspecific trait variation to total plant trait variation (Siefert *et al.* 2015), and from other studies that found that half of the variation in the LES is within-species variation (Fajardo and Siefert 2018). Intraspecific trait variation can also be substantial at regional scales in tropical and subtropical forests (Choat *et al.* 2007; Umaña and Swenson 2019) and temperate forests (Fajardo and Piper 2011; Hajek *et al.* 2013; Fajardo and Siefert 2018). However, our results contrast with those that have found that functional traits, especially root structural traits, are highly associated with phylogeny at levels above family (Valverde-Barrantes *et al.* 2017). The fact that we used a single genus may explain the discrepancy. Nevertheless, the implications of our results are profound, as high intraspecific trait variation can drive variation in whole-plant performance (Westerband *et al.* 2021), and this may help explain the patterns we observed in *Costus* species. High individual variation may result from species responses to environmental conditions (trait plasticity) or ecotypic differentiation of populations within species, as several species in our study are found at more than one site. A recent study of *Costus* across environmental gradients suggested that plasticity is one of the strongest drivers of trait–environment relationships (E. Ávila-Lovera *et al.,* submitted for publication). Regardless of the mechanism, high individual variation highlights the ability of *Costus* species to adjust leaf, above-ground stem, rhizome and fine-root traits to match the local environmental conditions, which can help mediate responses to changes in climate. However, further studies are necessary to evaluate if high individual variation in physiology has fitness advantages (Nolting *et al.* 2020).

## Conclusions

We conclude that *Costus* species show two apparent trade-offs between resource acquisition and conservation, one relating to water use and one to the LES. Taken together, these axes determine a unified whole-plant functional strategy for each *Costus* species. There was correlated evolution among multiple traits, especially those related to water movement and use. Finally, there was little variation in traits ascribed to site, but high individual variation in most traits, indicating high within-site and within-species variation.

## Supporting Information

The following additional information is available in the online version of this article—

**Figure S1.** Phylogenetic tree used to perform phylogenetic independent contrasts analysis and phylogenetic signal tests. Numbers following species names are unique identifiers (the species names match those in Vargas *et al.* 2021).

**Figure S2.** Principal components analysis (PCA) biplots using individual data points grouped by (A) species, and (B) site. Species and site abbreviations are as shown in Table 1.

**Figure S3.** Individual values of chlorophyll concentration by species and site. Abbreviations are as in Table 1 and Fig. 2.

**Figure S4.** Individual values of stomatal conductance by species and site. Abbreviations are as in Table 1 and Fig. 2.

**Figure S5.** Individual values of leaf thickness by species and site. Abbreviations are as in Table 1 and Fig. 2.

**Figure S6.** Individual values of leaf area by species and site. Abbreviations are as in Table 1 and Fig. 2.

**Figure S7.** Individual values of leaf dry matter content by species and site. Abbreviations are as in Table 1 and Fig. 2.

**Figure S8.** Individual values of specific leaf area by species and site. Abbreviations are as in Table 1 and Fig. 2.

**Figure S9.** Individual values of lamina dry mass to petiole dry mass ratio by species and site. Abbreviations are as in Table 1 and Fig. 2.

**Figure S10.** Individual values of leaf area mass to petiole dry mass ratio by species and site. Abbreviations are as in Table 1 and Fig. 2.

**Figure S11.** Individual values of leaf phosphorus concentration by species and site. Abbreviations are as in Table 1 and Fig. 2.

**Figure S12.** Individual values of leaf potassium concentration by species and site. Abbreviations are as in Table 1 and Fig. 2.

**Figure S13.** Individual values of leaf carbon isotope composition by species and site. Abbreviations are as in Table 1 and Fig. 2.

**Figure S14.** Individual values of leaf carbon concentration by species and site. Abbreviations are as in Table 1 and Fig. 2.

**Figure S15.** Individual values of leaf nitrogen isotope composition by species and site. Abbreviations are as in Table 1 and Fig. 2.

**Figure S16.** Individual values of leaf nitrogen isotope composition by species and site. Abbreviations are as in Table 1 and Fig. 2.

**Figure S17.** Individual values of stem specific density by species and site. Abbreviations are as in Table 1 and Fig. 2.

**Figure S18.** Individual values of rhizome water content by species and site. Abbreviations are as in Table 1 and Fig. 2.

**Figure S19.** Individual values of rhizome specific density by species and site. Abbreviations are as in Table 1 and Fig. 2.

**Figure S20.** Individual values of specific root length by species and site. Abbreviations are as in Table 1 and Fig. 2.

**Figure S21.** Individual values of fine-root diameter by species and site. Abbreviations are as in Table 1 and Fig. 2.

**Figure S22.** Individual values of root tissue density by species and site. Abbreviations are as in Table 1 and Fig. 2.

**Table S1.** Data set used for the analyses of this article (separate excel file).

**Table S2.** Table showing values of Blomberg’s *K* and *P*-values associated with the test of H_0_: *K* = 0. *P-*values lower than 0.1 are in italics.

**Table S3.** Correlation coefficients and *P*-values for the cross-species correlations and the correlations using phylogenetic independent contrasts (PICs) (separate excel file).

**Notes S1.** Research permit information.

plab073_suppl_Supplementary_Figure_S1

plab073_suppl_Supplementary_Figure_S2

plab073_suppl_Supplementary_Figure_S3

plab073_suppl_Supplementary_Figure_S4

plab073_suppl_Supplementary_Figure_S5

plab073_suppl_Supplementary_Figure_S6

plab073_suppl_Supplementary_Figure_S7

plab073_suppl_Supplementary_Figure_S8

plab073_suppl_Supplementary_Figure_S9

plab073_suppl_Supplementary_Figure_S10

plab073_suppl_Supplementary_Figure_S11

plab073_suppl_Supplementary_Figure_S12

plab073_suppl_Supplementary_Figure_S13

plab073_suppl_Supplementary_Figure_S14

plab073_suppl_Supplementary_Figure_S15

plab073_suppl_Supplementary_Figure_S16

plab073_suppl_Supplementary_Figure_S17

plab073_suppl_Supplementary_Figure_S18

plab073_suppl_Supplementary_Figure_S19

plab073_suppl_Supplementary_Figure_S20

plab073_suppl_Supplementary_Figure_S21

plab073_suppl_Supplementary_Figure_S22

plab073_suppl_Supplementary_Table_S1

plab073_suppl_Supplementary_Table_S2

plab073_suppl_Supplementary_Table_S3

plab073_suppl_Supplementary_Notes_S1

## Data Availability

All the trait data can be found in **Supporting Information—Table S1**. Additionally, data and R codes can be found in 10.5281/zenodo.5639572.

## Notes

**Form & Function. Chief Editor**: Kate McCulloh
