# Supplementary material for: Above- and below-ground functional trait coordination in the Neotropical understory genus Costus
Source: AoB Plants. 2021 Dec 2;14(1):plab073. doi: 10.1093/aobpla/plab073 (PMC8757582; doi:10.1093/aobpla/plab073)
Supplement: plab073_suppl_Supplementary_Figure_S1 [file plab073_suppl_supplementary_figure_s1.docx]

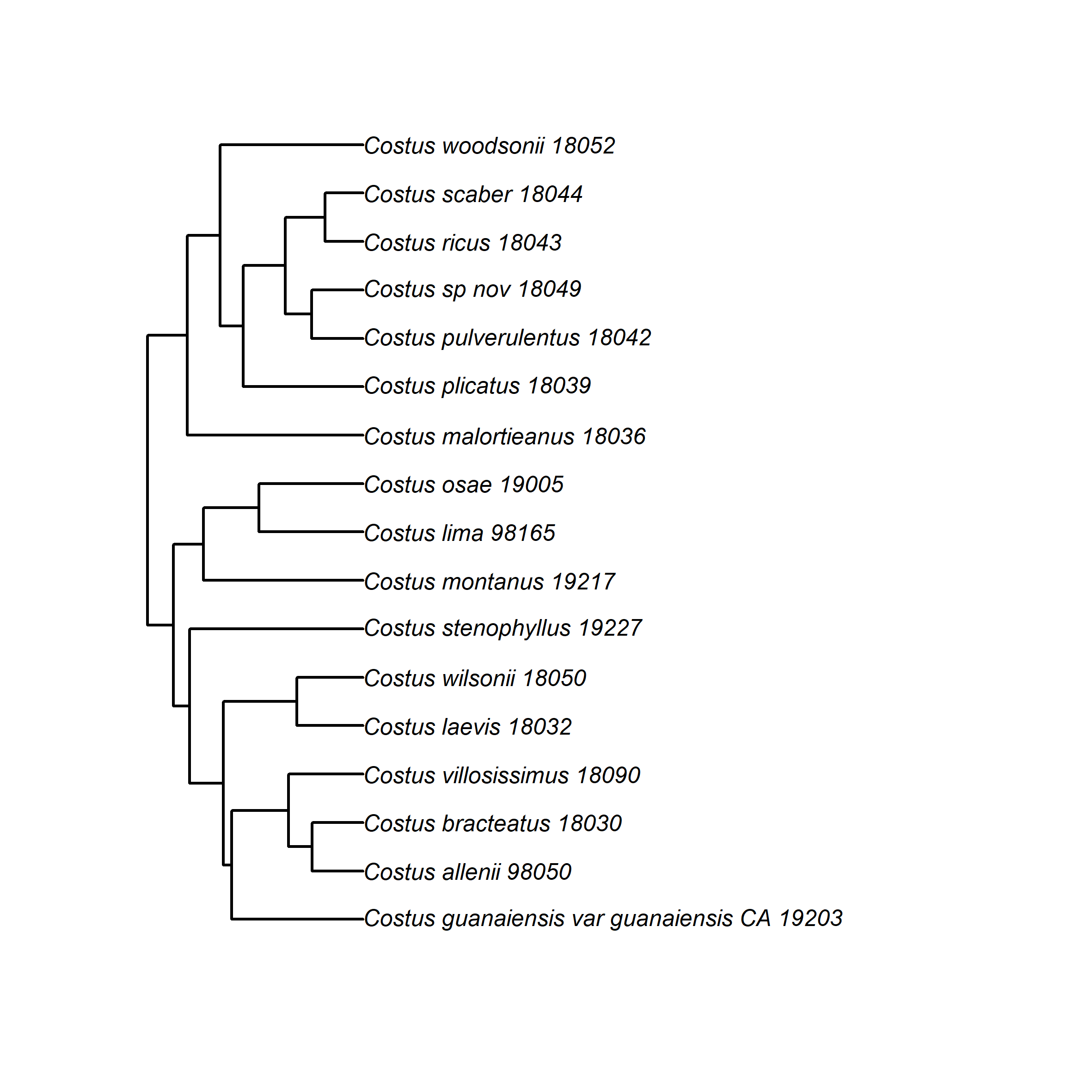


**Fig. S1** Phylogenetic tree used to perform phylogenetic independent contrasts analysis and phylogenetic signal tests. Numbers following species names are unique identifiers (the species names match those in Vargas et al. 2021).
