# Supplementary material for: Above- and below-ground functional trait coordination in the Neotropical understory genus Costus
Source: AoB Plants. 2021 Dec 2;14(1):plab073. doi: 10.1093/aobpla/plab073 (PMC8757582; doi:10.1093/aobpla/plab073)
Supplement: plab073_suppl_Supplementary_Figure_S2 [file plab073_suppl_supplementary_figure_s2.docx]

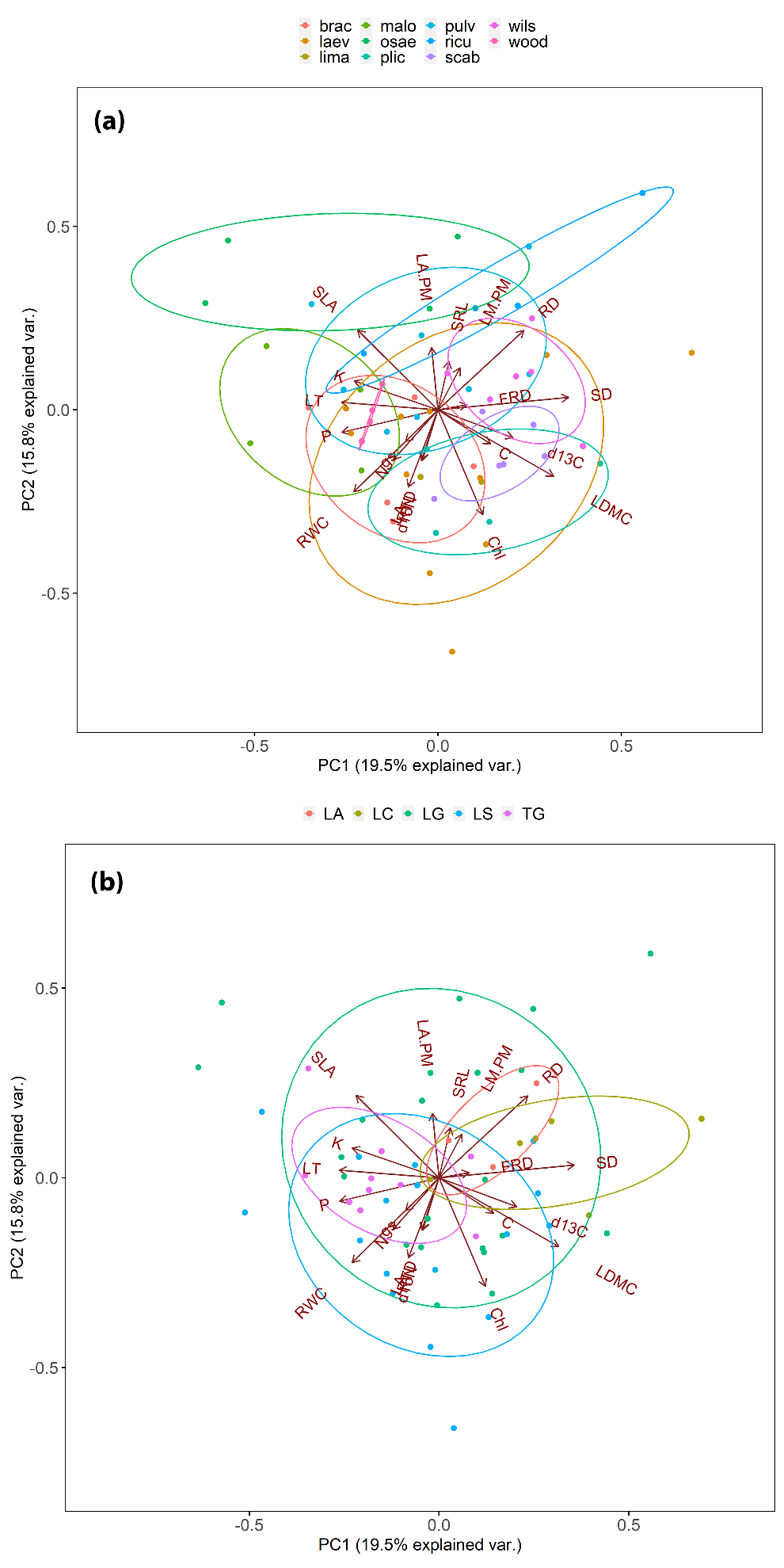


**Fig. S2** PCA biplots using individual data points grouped by (a) species, and (b) site. Species and site abbreviations are as shown in Table 1.
