# Supplementary figures and images for: Above- and below-ground functional trait coordination in the Neotropical understory genus Costus
Source: AoB Plants. 2021 Dec 2;14(1):plab073. doi: 10.1093/aobpla/plab073 (PMC8757582; doi:10.1093/aobpla/plab073)

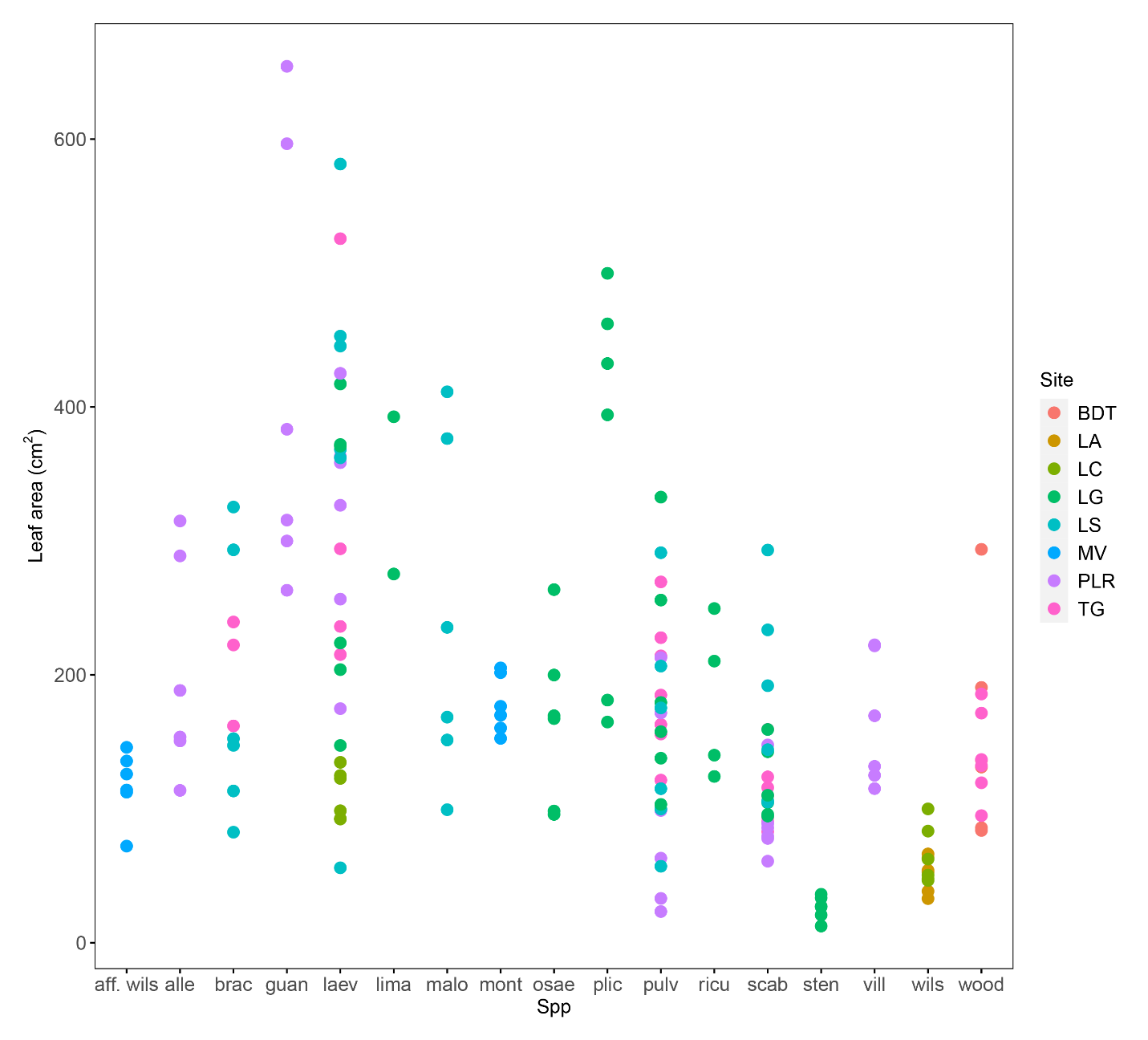


**Fig. S6** Individual values of leaf area by species and site. Abbreviations are as in Table 1 and Fig. 2.

Supplement: plab073_suppl_Supplementary_Figure_S6 [file plab073_suppl_supplementary_figure_s6.docx]
