# Supplementary material for: Above- and below-ground functional trait coordination in the Neotropical understory genus Costus
Source: AoB Plants. 2021 Dec 2;14(1):plab073. doi: 10.1093/aobpla/plab073 (PMC8757582; doi:10.1093/aobpla/plab073)
Supplement: plab073_suppl_Supplementary_Figure_S12 [file plab073_suppl_supplementary_figure_s12.docx]

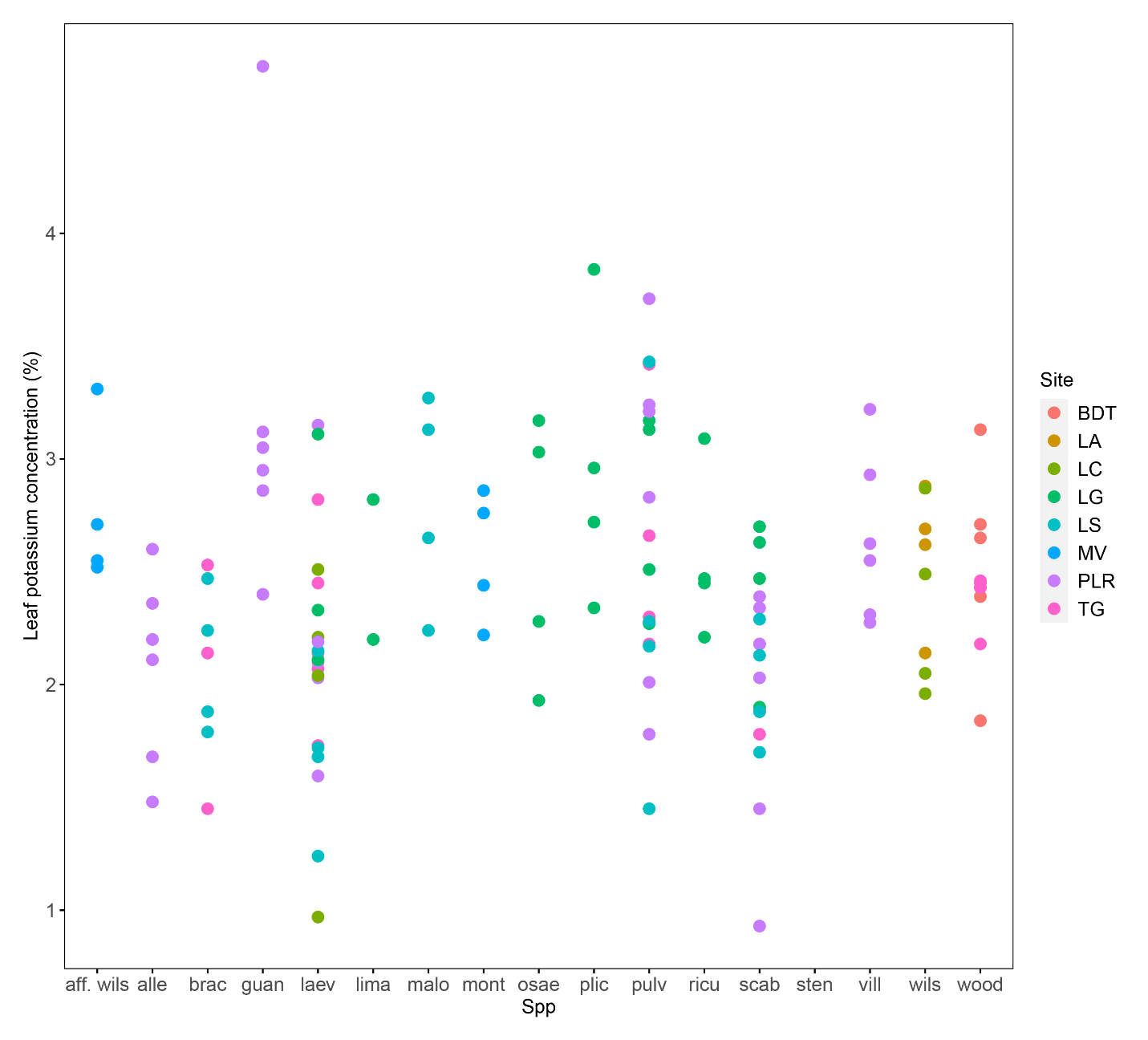


**Fig. S12** Individual values of leaf potassium concentration by species and site. Abbreviations are as in Table 1 and Fig. 2.
