# Supplementary material for: Above- and below-ground functional trait coordination in the Neotropical understory genus Costus
Source: AoB Plants. 2021 Dec 2;14(1):plab073. doi: 10.1093/aobpla/plab073 (PMC8757582; doi:10.1093/aobpla/plab073)
Supplement: plab073_suppl_Supplementary_Table_S2 [file plab073_suppl_supplementary_table_s2.docx]

**Table S2** Table showing values of Blomberg’s K and *P* values associated with the test of H_o_: K = 0. Trait abbreviations are as shown in Table 2. *P* values lower than 0.1 are in italics.

| Trait | Blomberg’s K | *P* |
| --- | --- | --- |
| Chl | 0.46 | 0.788 |
| g_s_ | 0.50 | 0.728 |
| LT | 0.65 | 0.271 |
| LA | 0.65 | 0.339 |
| LDMC | 0.62 | 0.368 |
| SLA | 0.59 | 0.447 |
| LM:PM | 0.73 | 0.154 |
| LA:PM | 0.56 | 0.598 |
| P | 0.50 | 0.699 |
| K | 0.62 | 0.393 |
| δ^13^C | 0.60 | 0.411 |
| C | 0.64 | 0.331 |
| δ^15^N | 0.65 | 0.377 |
| N | 0.39 | 0.934 |
| SSD | 0.59 | 0.470 |
| RhWC | 0.81 | *0.063* |
| RhSD | 0.81 | *0.077* |
| SRL | 0.38 | 0.951 |
| FRD | 0.64 | 0.336 |
| RTD | 0.37 | 0.951 |
