# Supplementary material for: Above- and below-ground functional trait coordination in the Neotropical understory genus Costus
Source: AoB Plants. 2021 Dec 2;14(1):plab073. doi: 10.1093/aobpla/plab073 (PMC8757582; doi:10.1093/aobpla/plab073)
Supplement: plab073_suppl_Supplementary_Notes_S1 [file plab073_suppl_supplementary_notes_s1.docx]

**Above- and below-ground functional trait spectra are weakly coordinated in Neotropical understory herbs**

**Notes S1** Research permit information.

All research was conducted with appropriate collection permits in Costa Rica (M-P-SINAC-PNI-ACAT-026-2018, ACC-PI-027-2018, M-PC-SINAC-PNI-ACLAP-020-2018, INV-ACOSA-076-18, M-PC-SINAC-PNI-ACTo-020-18) and Panama (SE/AP-13-19), and USDA import permits (USDA APHIS P.P.Q. P37-17-01100).
